# The predictive role of the total potassium intake and odds of breast cancer: a case-control study

**DOI:** 10.1186/s12885-024-12769-7

**Published:** 2024-08-12

**Authors:** Hamid Ahmadirad, Mostafa Norouzzadeh, Farshad Teymoori, Mitra Kazemi Jahromi, Hossein Farhadnejad, Mitra Babrpanjeh, Ebrahim Mokhtari, Zeinab Heidari, Parvin Mirmiran, Bahram Rashidkhani

**Affiliations:** 1 Student Research Committee, Nutrition and Endocrine Research Center, Research Institute for Endocrine Sciences, Shahid Beheshti University of Medical Sciences, Tehran, Iran; 2 Nutrition and Endocrine Research Center, Research Institute for Endocrine Sciences, Shahid Beheshti University of Medical Sciences, Tehran, Iran; 3 Nutritional Sciences Research Center, Iran University of Medical Sciences, Tehran, Iran; 4 Department of Nutrition, School of Public Health, Iran University of Medical Sciences, Tehran, Iran; 5 Endocrinology and Metabolism Research Center, Hormozgan University of Medical Sciences, BandarAbbas, Iran; 6 Department of Community Nutrition, Faculty of Nutrition Sciences and Food Technology, National Nutrition and Food Technology Research Institute, Shahid Beheshti University of Medical Sciences, Tehran, Iran; 7 Department of Community Nutrition, School of Nutrition and Food Sciences, Shiraz University of Medical Sciences, Shiraz, Iran; 8 Student Research Committee, Ahvaz Jundishapur University of Medical Sciences, Ahvaz, Iran

**Keywords:** Potassium intake, Breast cancer, Women, Iran, Case-control study

## Abstract

**Backgrounds:**

Dietary potassium can play an important role in decreasing inflammatory factors as a protective factor for cancers. In this case-control study, we aimed to assess the possible association between dietary potassium intake and the risk of breast cancer (BC) among Iranian adult women.

**Methods:**

The present case-control study was conducted at Shohada and Imam Hossain hospitals, in Tehran. The study included 134 newly diagnosed cases of BC and 267 controls. A validated semi-quantitative 168-item food frequency questionnaire was used to compute the potassium intake. Logistic regression, adjusted for potential confounders, was used to estimate odds ratios(ORs) and 95% confidence intervals(CI) of BC according to tertiles of potassium intake.

**Results:**

The mean(M) ± standard deviation(SD) of age and body mass index (BMI) were 47.9 ± 10.3 years and 29.4 ± 5.5 kg/m^2^, respectively. Also, the M ± SD of potassium intake for the control and case groups was 1616 ± 293 and 1542 ± 338 (mg/1000 Kcal), respectively. In the multivariable-adjusted model for potential confounders, the higher total potassium intake was associated with decreased odds of BC (OR: 0.35, 95%CI: 0.19–0.62, P for trend < 0.001). Moreover, an inverse relationship was observed between potassium from plant sources (OR: 0.39, 95%CI: 0.22–0.69, P for trend = 0.001) and fruit and vegetable sources (OR: 0.49, 95%CI: 0.28–0.87, P for trend = 0.016) and odds of BC.

**Conclusions:**

Our findings suggested that diet rich in potassium may have a predictive role to reduce the odds of BC.

**Supplementary Information:**

The online version contains supplementary material available at 10.1186/s12885-024-12769-7.

## Backgrounds

Breast cancer (BC) is considered the most common malignancy affecting women, with 2.3 million new cases accounting for 11.7% of the total cancer cases and the one of leading causes of cancer mortality with 685,000 deaths [1], and its incidence is 25 per 100,000 among Iranian women [2]. Age, genetic mutations, late pregnancy, hormone therapy, late menopause, premature menstruation, oral contraceptives, and cancer family history are well-known unmodifiable risk factors related to the development of BC [3], however, unhealthy diet is recognized as a modifiable risk factor in BC pathogenesis [4].

Many studies have suggested that dietary intake of different minerals such as calcium, iron, zinc, and selenium may affect BC risk [5–7]. A meta-analysis of prospective cohort studies demonstrated an inverse dose-response association between Ca intake and risk of BC [5]. Liu et al. revealed iron from white meat and plants was inversely associated with BC risk [6]. A case-control study of Chinese women observed a negative association between zinc from white meat and BC risk, however, there was a positive association between selenium from red meat and risk of BC [7].

Potassium is one of the most important minerals in the human body and it is referred to as a dietary anticarcinogenic agent [8], which mainly was derived from fruits and vegetables [9]. Potassium has a beneficial effect on human health due to the reduced role of potassium in the acidity of the blood and maintaining the acid-base balance [10]. Recently, emerging evidence has revealed that dietary intake of potassium may show a protective effect against the risk of different cancers such as lung cancer [11], colorectal cancer [12,29], and gastric cancer [13]. Also, some studies have shown the indirect role of potassium intake as a negative component of dietary acid load in the prediction of BC risk [14–16]. However, there are few studies on the possible association between potassium intake and the risk of BC, which indicates controversial results. [17, 18]. The study conducted by Levi et al. indicated a significant inverse association between potassium intake and the risk of BC [18], however, another study did not find any significant relationship between dietary intake of potassium and the risk of BC [17].

Regarding the limiting evidence and controversial findings on the relationship between dietary intake of potassium and BC risk, we aimed to assess the possible link between dietary potassium intake and odds of BC in a case-control design among Iranian women.

## Materials and methods

### Study population

This hospital-based case-control study was carried out among Iranian women, aged 30 to 65 years old, at Imam Hossain and Shohada hospitals, in Tehran (the capital of Iran) between September 2015 and February 2016. The case group consisted of 136 women with BC who were newly (within the past 6 months) diagnosed with histologically confirmed. At the same time, the control group consisted of 272 women were selected among people admitted to these 2 referral hospitals for a wide spectrum of non-neoplastic diseases that were unrelated to alcohol consumption, smoking, and long-term diet modification. Imam Hossein and Shohada Hospitals are general and main reference hospitals in the area covered by Shahid Beheshti University in Tehran, so many cases and controls from different parts of the country refer to these two hospitals. There was no statistically significant difference between the case and control groups in terms of living in Tehran or other parts of the country (*P*-value = 1.00) also, in different parts of the Tehran city (*P*-value = 0.545). So, the cases and controls are not representative of a specific population group and there is no difference between them in terms of geography and ethnicity.

Controls were matched to cases on age (within 5 years). Conditions in the control group included traumas and orthopedic disorders, disk disorders, acute surgical conditions, and eye, ear, nose, or skin disorders. The participation rate of the study was 92% and approximately %8 of the study population refused the interview. Seven participants were excluded from this analysis due to their reported energy intakes being outside of the range of ± 3 standard deviation (SD) from the mean (M) energy intakes of the study population (*n* = 2 cases, 5 controls). Finally, 401 eligible subjects included in the final analysis (134 cases and 267 controls).

The ethics research committee approved the study’s protocol of the Student Research Committee, Shahid Beheshti University of Medical Sciences, Tehran, Iran (ethical approval code: IR.SBMU.RETECH.REC.1402.729). The written informed consent form was obtained from all participants before enrolment into the study.

### Dietary assessment

The trained dietitian interviewers collected the dietary data of all participants using a validated semi-quantitative 168-item food frequency questionnaire (FFQ) [19]. In this way, the frequency of food intake during one year before the time of the interview was evaluated.

The evaluation of the validity and reliability results of the FFQ used in the present study regarding the estimation of potassium intake showed a strong reliability of the FFQ in estimating the potassium intake of men (*r* = 0.81) and women (*r* = 0.77). Also, regarding the validity of potassium intake, which compared the FFQ used in our study with 12 24-hour recalls and examined the correlation of estimated potassium intake with FFQ and 12 recalls, which were 0.33 and 0.31 for men and women, respectively. The matching percentage of people in terms of placement in the same class between FFQ and 24-hour recall is 50 and 40%, respectively. From the point of view of validity, due to the high dispersion in food intakes, the obtained values ​​are acceptable and reasonable [19].

Individuals were asked to express dietary intake of all food items based on daily, weekly, and monthly frequency then we used the standard Iranian household measures to convert the frequencies to daily grams of food intake [20]. Daily energy and nutrient intake were computed using the United States Department of Agriculture’s (USDA) food composition Table [21]. The Iranian food composition table was used for traditional foods which were not available in the USDA database. To compute the total potassium intake, amounts of dietary potassium consumed from each 168-food item of FFQ were summed up. To calculate the amount of dietary potassium intake from plant and animal sources, the potassium value of each food item in the plant and animal sources is summed, separately.

### Anthropometric and physical activity measurements

Anthropometric assessment was done by a trained dietician. Participants’ weight was measured using a digital scale (Seca, Hamburg, Germany) while they were minimally clothed and without shoes. The accuracy of weight measurement was 0.5 kg. To measure the height of the individuals, we used a non-elastic tape meter fixed to a wall with no shoes to the nearest 0.5 cm. Body mass index (BMI) was computed as weight (kg) divided by the square of height (meter). A non-stretchable tape (with an accuracy of up to 0.5 cm) was used to measure waist circumference (WC) (at the level midway between the lowest rib margin and the iliac) and hip circumference (HC) (at the widest point over the buttocks) without exerting any pressure on body skin of participants. Subsequently, the waist-to-hip ratio (WHR) was calculated as WC (in cm) divided by HC (in cm).

Data on the participant’s physical activity (PA) was assessed using the International Physical Activity Questionnaire (IPAQ) through face-to-face interviews [22]. IPAQ is a set of questionnaires used to provide a common instrument that can be used to obtain internationally comparable data on health–related PA. It is available in both long and short versions. We used the short form in the current work. The IPAQ short form asks about three specific types of activity undertaken in the last 7 days: walking, moderate-intensity activities, and vigorous-intensity activities. Responses are used to estimate the volume of PA according to the following formula: [Total physical activity = Walk + Moderate + Vigorous]. The total score is expressed as MET-minutes/week [23, 24].

### Assessment of other variables

Information on participants’ socio-demographics, lifestyle, and clinical including, age (years), age at menarche (years), age at first pregnancy (years), menopausal status (pre-menopause, post-menopause), education (illiterate, less than a high school diploma, high school diploma and more), cancer family history (yes = 1, no = 0), BC family history (yes = 1, no = 0), marital status (single, married, divorced, widowed), smoking status (yes = 1, no = 0), supplement intakes over the last year (including vitamin A, vitamin D, vitamin E, B complex, folic acid, Vitamin C, β carotene, calcium, iron, zinc, selenium, multivitamins-minerals, omega-3 fatty acids, and probiotics) (yes = 1, no = 0; If yes, the complementary information on dose and frequency), and anti-inflammatory drug use (yes = 1, no = 0) was collected using general questionnaires.

### Statistical analysis

Data were analyzed using the Statistical Package Software for Social Science, version 21 (SPSS Inc., Chicago, IL, USA). We used the histogram chart and Kolmogorov-Smirnoff test to examine the normality of variables. Information on participants’ characteristics and dietary intakes was expressed as M ± SD and frequency (percentages) for quantitative and qualitative variables, respectively. To compare the difference in categorical and continuous variables between cases and controls, a chi-square test and an independent sample t-test were used, respectively. The participants were categorized into tertile according to the total potassium intake and potassium from plant and animal food sources. We used linear regression and the chi-square test to calculate the P-trend of the continuous and categorical variables. P-trend calculation was done by entering the variable “median potassium intake” in each of the tertiles in the logistic regression model and calculating the resulting *p*-value.

The odds ratios (ORs) and 95% confidence intervals (95% CIs) of BC across tertiles of the total potassium intake and potassium from plant and animal food sources were determined by logistic regression analysis in crude model and adjusted model for various potential confounders including age, menopausal status, family history of cancer, anti-inflammatory drug use, vitamin D supplementation, PA, BMI, and energy intake. regarding smoking, considering that only 3% of the population participating in this study smoked, and on the other hand, there was no significant difference between the case and control groups in terms of smoking (*P*-value = 0.842), so it has not been adjusted to reduce the number of input variables to the logistic model of smoking. Also, adjusting for smoking did not change the results, so we did not include it in the models. A *P*-value of < 0.05 was considered statistically significant. Dose-response analysis was conducted using probit regression analysis. Dietary intake of potassium (500 mg) as the independent variable (dose) and BC status (yes/no) as the response variable were included in this analysis.

## Results

The M ± SD of age of participants was 49.5 ± 10.7 and 47.1 ± 10.0 years in cases and controls, respectively. Also, the M ± SD of potassium intake for the control and case groups was 1616 ± 293 and 1542 ± 338 (mg/1000 Kcal), respectively.

**Fig. 1:**
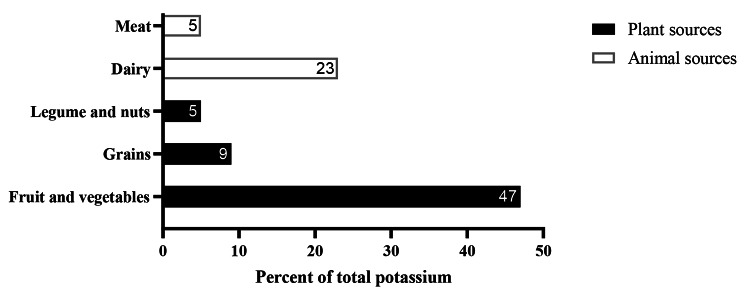
Distribution of dietary potassium intake from the main food sources

As Fig. 1 shows, 69.6% of total potassium intake in the study population was derived from plant sources including fruit-vegetables (47.1%), grains (9.3%), and legume-nuts (5.6%), respectively. Potassium intakes from the animal source including dairy (23.6%) and meat (5.3%) constitute 29.9% of total potassium intake. The distribution of dietary potassium intake (as mg and percentage) from main sources is indicated in Supplementary Table 1.

Table 1 shows the data on the socio-demographic, anthropometric, lifestyle characteristics, and dietary intake of the study population in case and control groups. The M of age (*P*-value = 0.035), % of menopausal status (*P*-value = 0.037), and % of cancer family history (*P*-value = 0.028) in participants with BC were significantly higher than the control group. However, % of participants who used anti-inflammatory drugs (*P*-value = 0.007) and had vitamin D supplement intake (*P*-value = 0.029) in the control group was significantly higher than the case group. The participants in the control group significantly consumed more potassium (*P*-value = 0.025) and energy intake (*P*-value = 0.015) compared to the case group. However, there were no significant differences in other dietary intakes, including macronutrients, plant sources, fruit and vegetables, grains, legumes and nuts, animal sources, dairy, and meat between cases and controls.

**Table 1:** Study population characteristics among the breast cancer patients and control group^1^

	Control (*n* = 267)	Case (*n* = 134)	*P*-value^2^
**Demographic data**			
Age (year)	47.1 ± 10.0	49.5 ± 10.7	0.035
Body mass index (Kg.m^2^)	29.0 ± 5.4	30.1 ± 5.7	0.071
Physical activity (MET/min/week)	32.7 ± 5.2	32.9 ± 5.4	0.701
Smoking (yes, %)	3.4	3	0.842
Menopausal status (yes, %)	42.7	53.7	0.037
Cancer family history (yes, %)	20.6	30.6	0.028
Inflammatory disease history (yes, %)	13.1	10.4	0.435
Comorbidities (yes, %)	37.1	38.1	0.891
Anti-inflammatory drug (yes, %)	17.2	7.5	0.007
Vitamin D supplement intake (yes, %)	24.3	14.9	0.029
**Dietary intakes**			
Energy intake (Kcal/d)	2753 ± 798	2562 ± 612	0.015
Carbohydrate (% of energy)	53.0 ± 6.4	52.4 ± 6.1	0.437
Protein (% of energy)	12.7 ± 2.1	12.4 ± 2.0	0.100
Fat (% of energy)	34.3 ± 6.7	35.2 ± 6.6	0.213
Potassium (mg/1000 Kcal)	1616 ± 293	1542 ± 338	0.025
**Potassium from food sources (mg/1000Kcal)**			
Plant sources	1111 ± 218	1542 ± 338	0.105
Fruit and vegetables	763 ± 208	735 ± 255	0.247
Grains	142 ± 70	138 ± 59	0.579
Legume and nuts	92.1 ± 59.4	82.6 ± 58.6	0.132
Animal sources	497 ± 238	465 ± 211	0.189
Dairy	400 ± 235	366 ± 211	0.157
Meat	83.3 ± 37.7	83.2 ± 43.3	0.975

^1^Data are expressed as mean ± SD and percent (%) for continuous and categorical variables, respectively

^2^*P*-values were computed using the independent sample t-test and chi square for continues and categorical variables, respectively

Table 2 indicates the characteristics of the study participants across the tertiles of potassium intake (per 1000 Kcal). There were no significant differences in age, BMI, PA, menopausal status, cancer family history, anti-inflammatory drug, and vitamin D supplement intake among participants across the tertiles of potassium intake. However, dietary intakes of protein, potassium, dietary potassium from plant sources, potassium from fruit-vegetables, legume-nuts, potassium from animal sources, and potassium from dairy significantly increased in participants across tertiles of potassium intake, whereas, dietary intake of energy, fat, and potassium from grain significantly decreased across tertiles of potassium intake.

**Table 2:** Study population characteristics based on the tertiles of potassium intake per 1000 kcal of energy intake according to the cut points of control group^1^

	Tertiles of potassium intake per 1000 Kcal			*P*-trend^2^
	T1(*n* = 153)	T1(*n* = 129)	T1(*n* = 119)	
**Demographic data**				
Age (year)	47.0 ± 10.0	48.3 ± 10.1	48.5 ± 10.8	0.216
Body mass index (Kg.m2)	29.6 ± 5.6	29.5 ± 5.6	29.0 ± 5.1	0.445
Physical activity (MET/min/week)	32.6 ± 4.6	32.1 ± 5.1	33.5 ± 5.9	0.217
Smoking (yes, %)	2.6	4.7	2.5	0.561
Menopausal status (yes, %)	49.6	45	45.1	0.707
Cancer family history (yes, %)	26.1	21.7	23.5	0.659
Anti-inflammatory drug (yes, %)	14.4	10.9	16.8	0.386
Vitamin D supplement intake (yes, %)	22.2	20.9	20.2	0.915
**Dietary intakes**				
Energy intake (Kcal/d)	2813 ± 820	2657 ± 698	2565 ± 674	< 0.001
Carbohydrate (% of energy)	53.6 ± 8.0	54.2 ± 6.1	54.4 ± 6.0	0.387
Protein (% of energy)	11.8 ± 1.9	12.9 ± 1.8	14.1 ± 1.9	< 0.001
Fat (% of energy)	37.4 ± 8.0	34.9 ± 5.8	33.3 ± 4.8	< 0.001
Potassium (mg/1000 Kcal)	1291 ± 142	1610 ± 74.7	1957 ± 207	< 0.001
**Potassium from food sources (mg/1000Kcal)**				
Plant sources	946 ± 153	1105 ± 171	1285 ± 257	< 0.001
Fruit and vegetables	602 ± 143	757 ± 139	944 ± 241	< 0.001
Grains	154 ± 77.5	138 ± 60.1	126 ± 56.4	0.001
Legume and nuts	78.2 ± 56.7	91.3 ± 59.9	100 ± 59.7	0.002
Animal sources	340 ± 133	498 ± 175	662 ± 252	< 0.001
Dairy	244 ± 129	397 ± 167	565 ± 255	< 0.001
Meat	78.6 ± 38.6	86.7 ± 39.6	85.5 ± 40.6	0.134

^1^Data are expressed as mean ± SD and percent (%) for continuous and categorical variables, respectively

^2^P-trend were computed using the linear regression and chi square for continues and categorical variables, respectively

The OR and 95% CI of BC across tertiles of total potassium intake and its food sources are presented in Table 3. In the crude model, the participants in the third tertile of total potassium intake had lower odds of BC than those in the first tertile (OR: 0.45, 95%CI: 0.27–0.77, P for trend = 0.003). Also, in the adjusted model, after adjustment for age, menopausal status, family history of cancer, anti-inflammatory drug use, vitamin D supplementation, PA, BMI, and energy intake, decreased the odds of BC in third tertiles of total potassium intake remained significant (OR: 0.35, 95%CI: 0.19–0.62, P for trend < 0.001). The odds of BC in participants in the highest tertile of potassium intake from plant sources were significantly lower than those in the lowest tertile based on the crude (OR: 0.49, 95%CI: 0.29–0.83, P for trend = 0.007) and adjusted model (OR: 0.39, 95%CI: 0.22–0.69, P for trend = 0.001). There was no significant association between potassium intake from animal sources and the odds of BC.

**Table 3:** The OR (95%CI) of breast cancer based on the total potassium intake and from its plant and animal food sources in tertiles or per increment of one standard deviation among study population

	OR of breast cancer (95% CI)					
	T1	T2	T3	*P* trend	Per one SD	*P*-value
**Total potassium (per 1000Kcal)**						
Median score, SD	1318	1572	1820	-		-
Case / total	64/153	40/129	30/119	-	134 / 401	-
Crude model	1.00 (Ref)	0.58 (0.35–0.96)	0.45 (0.27–0.77)	0.003	0.77 (0.62–0.96)	0.021
Adjusted model^*^	1.00 (Ref)	0.51 (0.30–0.86)	0.35 (0.19–0.62)	< 0.001	0.70 (0.55–0.88)	0.084
**Potassium from plant sources (per 1000Kcal)**						
Median score, SD	889	1090	1335	-		-
Case / total	64/152	38/128	32/121	-	134 / 401	-
Crude model	1.00 (Ref)	0.55 (0.33–0.91)	0.49 (0.29–0.83)	0.007	0.83 (0.67–1.03)	0.106
Adjusted model^*^	1.00 (Ref)	0.48 (0.28–0.81)	0.39 (0.22–0.69)	0.001	0.76 (0.60–0.96)	0.022
**Potassium from animal sources (per 1000Kcal)**						
Median score, SD	277	466	717	-		-
Case / total	51/140	46/135	37/126	-	134 / 401	-
Crude model	1.00 (Ref)	0.86 (0.52–1.42)	0.70 (0.41–1.17)	0.177	0.85 (0.68–1.06)	0.152
Adjusted model^*^	1.00 (Ref)	0.85 (0.49–1.47)	0.66 (0.38–1.15)	0.147	0.82 (0.65–1.04)	0.107

^*^ Adjusted model: adjusted for age, menopausal status, family history of cancer, anti-inflammatory drug use, vitamin D supplementation, physical activity, body mass index, and energy intake

Table 4 shows the association between potassium intake from its five main food groups and the odds of BC. In the crude models, no significant association was observed between potassium intake from fruit and vegetable sources and the odds of BC. However, after adjustment for age, menopausal status, family history of cancer, anti-inflammatory drug use, vitamin D supplementation, PA, BMI, and energy intake, the odds of BC in participants of third tertiles of potassium intake from fruit and vegetable was lower than those in the first tertile (OR: 0.49, 95%CI: 0.28–0.87, P for trend = 0.016).

**Table 4:** The OR (95%CI) of breast cancer based on the potassium intake from its five main food group sources of in tertiles or per increment of one standard deviation among study population

	OR of breast cancer (95% CI)					
Potassium (per 1000Kcal) from:	T1	T2	T3	*P*-trend	Per one SD	*P*-value
**Fruit and vegetables sources**						
Median score, SD	564.8	730.3	968.0	-		-
Case / total	52/140	48/137	34/124	-	134 / 401	-
Crude model	1.00 (Ref)	0.86 (0.52–1.41)	0.62 (0.37–1.05)	0.077	0.87(0.70–1.09)	0.237
Adjusted model*	1.00 (Ref)	0.70 (0.41–1.19)	0.49 (0.28–0.87)	0.016	0.79(0.63–1.00)	0.052
**Grain sources**						
Median score, SD	86.1	129.8	198.0	-		-
Case / total	44/133	52/138	38/130	-	134 / 401	-
Crude model	1.00 (Ref)	1.18 (0.71–1.96)	0.82 (0.48–1.39)	0.393	0.94(0.76–1.17)	0.603
Adjusted model*	1.00 (Ref)	1.06 (0.62–1.81)	0.77 (0.44–1.36)	0.329	0.94(0.74–1.18)	0.593
**Legume and nut sources**						
Median score, SD	42.2	73.9	140.9	-		-
Case / total	55/143	43/133	36/125	-	134 / 401	-
Crude model	1.00 (Ref)	0.75 (0.46–1.24)	0.62 (0.37–1.04)	0.083	0.84(0.66–1.05)	0.137
Adjusted model*	1.00 (Ref)	0.78 (0.46–1.33)	0.70 (0.40–1.22)	0.241	0.87(0.68–1.11)	0.287
**Dairy sources**						
Median score, SD	179.6	361.5	606.3	-		-
Case / total	50/139	47/135	37/127	-	134 / 401	-
Crude model	1.00 (Ref)	0.90 (0.55–1.49)	0.70 (0.42–1.18)	0.185	0.84(0.68–1.04)	0.125
Adjusted model*	1.00 (Ref)	0.96 (0.56–1.65)	0.72 (0.41–1.24)	0.224	0.82(0.65–1.04)	0.106
**Meat sources**						
Median score, SD	48.0	77.5	119.0	-		-
Case / total	47/135	45/134	42/132	-	134 / 401	-
Crude model	1.00 (Ref)	0.94 (0.56–1.57)	0.86 (0.51–1.44)	0.568	0.99(0.81–1.23)	0.992
Adjusted model*	1.00 (Ref)	0.83 (0.48–1.43)	0.69 (0.40–1.21)	0.208	0.93(0.74–1.17)	0.586

* Adjusted model: adjusted for age, menopausal status, family history of cancer, anti-inflammatory drug use, Vitamin D supplementation, physical activity, body mass index, and energy intake

**Fig. 2:**
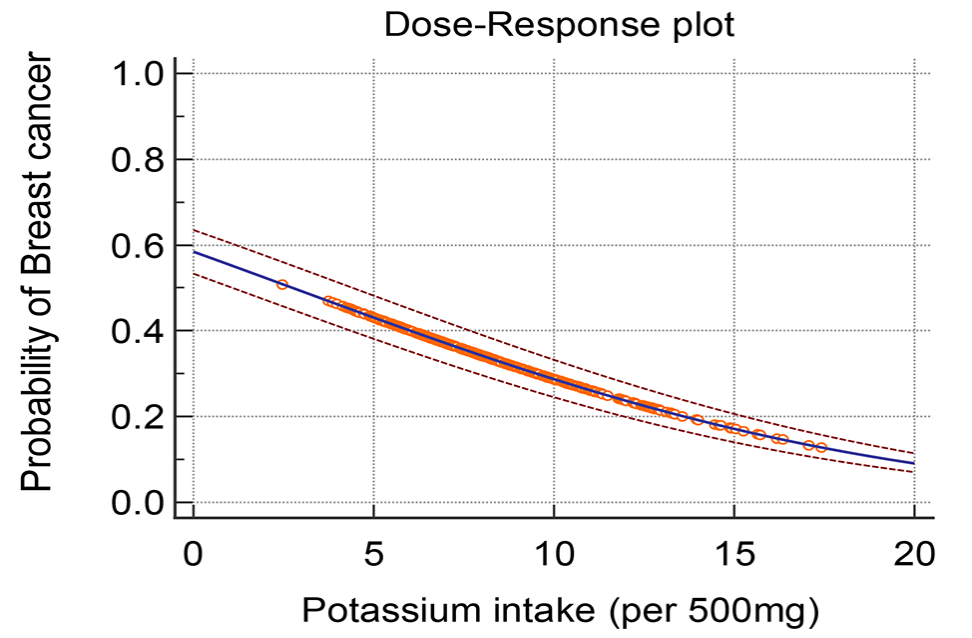
Dose-response plot for odds of breast cancer per increasing each 500 mg of potassium intake

We observed a dose-response association between potassium intake and the odds of BC. As Fig. 2 indicates by increasing each 500 mg of potassium intake, the odds of BC significantly decreased.

## Discussion

Our study findings showed that the higher intake of total potassium and the higher intake of potassium from plant sources were related to lower odds of BC. Also, there was a dose-response association between dietary potassium intake and BC risk, indicating that for each 500 mg increase in potassium consumption, the risk of developing BC decreases.

Although no epidemiological studies have investigated the relationship between total intake of potassium or intake of potassium from different food sources with the risk of BC, our findings are in line with the results of previous studies that reported that dietary potassium intake or consumption of foods rich in potassium can play a beneficial role in reducing the risk of chronic diseases. Our findings align with the World Health Organization’s recommendation to increase potassium intake to reduce the risk of chronic diseases [25]. Multiple studies have demonstrated an inverse association between dietary potassium intake and the risk of cardiovascular diseases and mortality [26, 27]. Belle et al. indicated that survivors of childhood cancer exhibited a reduced potassium intake, highlighting the importance of dietary advice for the enhancement of potassium consumption among childhood cancer survivors [28]. Another investigation suggested that dietary potassium was inversely associated with colorectal cancer risk [29]. Meanwhile, fruits, vegetables, and dairy are the primary contributors to potassium intake [30]. An inverse association has been observed between the consumption of non-starchy vegetables and the incidence of BC [31]. There is evidence suggesting that foods rich in potassium, such as tomatoes, bananas, and yogurt, may effectively reduce the incidence of lung cancer [32, 33]. While current guidelines recommend 400 g (5 servings) of fruits and vegetables daily for the general population, studies indicate that a higher intake is required to prevent cancer incidence. A dose-response meta-analysis revealed that consuming 550–600 g per day (7–7.5 servings) of vegetables and fruits is associated with a roughly 14% lower risk of total incident cancers [34]. Moreover, potassium intake has been shown to impact cancer prognosis, hence it is recommended that cancer survivors should have a higher intake of fruits and vegetables compared to the general population [35].

The mechanisms linking potassium intake to cancer risk remain unclear, however, a higher potassium intake is often accompanied by healthier dietary choices [30]. Antioxidants in fruits and vegetables help neutralize reactive oxygen species, reducing DNA damage [36], and induce detoxifying enzymes [37]. Fiber intake can influence steroid hormone concentrations and hormone metabolism [36], and positively impact gut microbiota [38]. A case-control study suggests that potassium intake may regulate TNF-a production, with women having the lowest risk for gastric cancer found among those homozygous for TNF-a variants with the highest potassium intake [39]. The regulatory impact of potassium on cellular proliferation could potentially yield anticancer properties through its influence on the folding and stabilization of G-quadruplexes, coupled with the inhibition of nuclear factor-κb expression [40]. An inadequate intake of potassium can disrupt the dietary acid balance, leading to low-grade systemic metabolic acidosis, a condition that tends to intensify with aging [41]. Also, lower potassium intake is often associated with higher dietary sodium consumption, creating an imbalance that can pose a risk for chronic diseases [42]. Notably, substances with carcinogenic properties decrease potassium concentration and elevate sodium concentration in cells, while anticarcinogenic agents produce the opposite effect. Dietary factors studied for their carcinogenic potential including sodium, fat, and calories. Conversely, recognized dietary anticarcinogenic factors include potassium and fiber [8].Even though the association between dietary potassium and serum potassium levels is not clearly understood, studies indicate that alterations in serum potassium levels may partially contribute to the effects of dietary potassium on cancer pathogenesis. In a case-control study, it is noted that there is a direct association between higher fasting levels of serum potassium in control group and an increased risk of cancer [43]. You et al., indicated that appropriate intake of potassium has a protective effect against lung cancer, however, with exceeded intake of potassium than the daily requirement, the protective effect would be weakened [11]. A proposed mechanism involves the inhibition of T-cell function against cancer cells. Elevated extracellular potassium concentrations lead to increased intracellular potassium in T-cells, impairing receptor phosphorylation and significantly suppressing T-cell effector function [44, 45]. Of note, the higher serum potassium levels among cancer patients can be attributed to the release of potassium from the necrosis of cancer cells [46].

The current study possesses notable strengths. To the best of our knowledge, this study is the first to examine the association between total potassium intake and the odds of BC. Also, a dose-response analysis was conducted to understand the shape of the association between potassium intake and BC risk. Furthermore, the study investigated the association between potassium intakes from both plant and animal sources and the risk of BC, providing further insights into the impact of the dietary source of potassium on the development of BC. Dietary data collection was accomplished through a validated and reproducible FFQ, administered by trained dietitians in face-to-face interviews, reducing the risk of measurement bias associated with self-reported questionnaires.

However, it is important to acknowledge some limitations. The case-control design of this study prohibits the establishment of causality between exposure and outcome. Additionally, in this case-control study, cases and controls were not matched based on key variables like menopausal status. This decision aimed to simplify the selection of eligible individuals and avoid prolonging the sampling time. However, the potential confounding influence of this variable was addressed in the multivariable-adjusted analysis. Moreover, FFQs are widely used in epidemiological studies to assess dietary intake and its relationship to health outcomes. Although, FFQs have some inherent limitations that cannot be neglected including recall bias, limited ability to capture non-dietary factors and etc. however we tried to minimize the limitations and shortcomings of this questionnaire by using a valid and reliable FFQ to have a proper estimate of the food intake of the participants. Also,  IPAQ is suitable for measuring recent PA and has low validity for evaluating lifetime PA. However, we did not have any other valid questionnaire and that is why we used this questionnaire. finally, some inherent limitations of the case-control study design including selection bias, information bias in determining exposure or outcome, and failure in elimination of the effect of all possible confounders should be considered.

## Conclusions

In conclusion, the results of the current study revealed that individuals with a diet rich in potassium may be less prone to BC odds. Future studies are needed to confirm these results and elucidate the association between dietary and serum levels of potassium, and the optimal potassium intake in both pre- and post-diagnosis cancer patients.

### Electronic supplementary material

Below is the link to the electronic supplementary material.

Supplementary Material 1

## Data Availability

The data analyzed in the present study are available by the corresponding author on a reasonable request.
