# Supplementary material for: The predictive role of the total potassium intake and odds of breast cancer: a case-control study
Source: BMC Cancer. 2024 Aug 12;24:995. doi: 10.1186/s12885-024-12769-7 (PMC11321017; doi:10.1186/s12885-024-12769-7)
Supplement: Supplementary file 1 — Supplementary Material 1 [file 12885_2024_12769_MOESM1_ESM.docx]

**Supplementary Table 1.** The main food sources of potassium intake among all participants.

|  | **mg intake of potassium** | **Percent of total potassium** |
| --- | --- | --- |
| **Plant sources** | 2903 ± 988 | 69.6 |
| Fruit and vegetables | 1982 ± 756 | 47.1 |
| Grains | 379 ± 223 | 9.3 |
| Legume and nuts | 240 ± 182 | 5.6 |
| **Animal sources** | 1279 ± 687 | 29.9 |
| Dairy | 1025 ± 665 | 23.6 |
| Meat | 215 ± 112 | 5.3 |

**Abbreviation:** mg, milligrams.
